# Testimonials

**Published:** 1910-07-09

**Authors:** 


### TESTIMONIALS.

It is quite an education to read testimonials: they are indeed human documents. They not only unfold the life story and character of the recipient, but also recall the manner of the giver. Unfortunately they do not always "proclaim the man" with the same degree of accuracy as Shakespeare ascribes to apparel; moreover it can hardly be said that their "quality is not strained" nor yet do they always "bless him who gives nor him who takes." Certainly the writing of testimonials is not an easy matter, especially in those frequently occurring cases when the subject is a man whose connection with the writer has been but slender or took place years ago. Then it is we come across the more or less ambiguous expressions such as "was an easy man to work with," was "well received by my patients." Many testimonials defeat their object by extravagant wording; even those in high places at the present day have been known to sin in this respect; curiously enough the surgeons seem to be more often guilty than the physicians. Of course, to some extent, a testimonial is to be regarded as an advertisement, and some latitude must be allowed in order to avoid any suggestion of "damning with faint praise," but references to "profound knowledge," "unremitting solicitude," and a frequent use of the superlative do depreciate greatly the value of the testimonial. Under present conditions the testimonial system still flourishes, and is, perhaps, a necessary evil; there is no doubt that it often becomes an unmitigated nuisance. What with the applications from newly-qualified men, almost forgotten old students, nurses, and sisters, one can easily imagine that a popular member of the staff of a hospital may well wish for some other less tiresome method of recording his opinion. It has been suggested that some sort of schedule, such as is in common use in Germany, might be made applicable to junior medical men. It would form a permanent record and be endorsed suitably at the end of each appointment by members of the staff or by the principal of a private practice.

A word of warning may be given to intending applicants for testimonials: do not ask for "just a few words" directly after passing the final; wait until there is a definite object in view. Sometimes men ask for testimonials before they have decided what to do; they seem to regard the possession of a collection as a fitting way to round off their school career. These testimonials are the most difficult to write and are the least valuable. But, above all, they are often unused while they are recent on account of the owner taking a house appointment in his own hospital or locums, or getting some work through friends, and so a great amount of trouble is caused unnecessarily. Later on they are not wanted because they do not mention what the applicant has done since qualification, and he does not like to bother again an influential member of the staff, whom he has already "tapped" once. A testimonial containing the name of the appointment sought after is more valuable than one only referring in general terms to a post as resident medical officer, etc.; but the latter is of course much more convenient if the testimonials are to be printed and if the owner of them is likely to be applying for several appointments within a year or two.

